# Emergency Caesarean Section: Influences on the Decision-to-Delivery Interval

**DOI:** 10.1155/2011/640379

**Published:** 2011-07-13

**Authors:** Aiste Cerbinskaite, Sarah Malone, Jennifer McDermott, Andrew D. Loughney

**Affiliations:** Women's Services, Royal Victoria Infirmary, Richardson Road, Newcastle upon Tyne NE1 4LP, UK

## Abstract

RCOG/NICE guidelines recommend that, for fetal compromise in labour, delivery should be accomplished ideally within 30 minutes. In this study, we investigated the factors which affect the decision-to-delivery (DD) intervals for emergency caesareans. To achieve this, prospective data were collected for all grade 1 and 2 caesareans performed on a busy labour ward over 12 months. We found that the ratio of labouring women to midwives had a significant effect on the DD intervals, which were significantly prolonged when 1 : 1 care was not provided (*P* < 0.001). The observed effect resulted exclusively from a prolonged transfer time to theatre. General anesthesia use shortened the DD interval for grade 1 caesareans (*P* < 0.001) and was more likely to be used during the day shift (*P* < 0.009). We conclude that midwifery staffing levels and the form of anaesthesia employed influence on DD intervals for the most urgent caesarean sections.

## 1. Introduction

In keeping with the recommendations of the National Confidential Enquiry into Patient Outcome and Death (NCEPOD), a four-step classification system for the urgency of caesarean sections has been adopted in many UK obstetric units. In this scheme, grade 1 caesareans are performed when there is an immediate threat to the life of the woman or fetus, grade 2 when there is evidence of maternal or fetal compromise which is not immediately life threatening, and grade 3 when there is no maternal or fetal concern but early delivery is required. Grade 4 caesareans are elective cases [1, 2]. Studies have demonstrated that for grade 1 and grade 2 caesareans, maternal and perinatal outcomes deteriorate measurably when the decision-to-delivery interval exceeds 75 minutes [3–6], and it is reasonable to suppose that delivery in a much shorter time frame would be required to preserve perinatal health in the most urgent situations. The National Institute for Health and Clinical Excellence (NICE) therefore recommends that in cases of confirmed or suspected acute fetal compromise, loosely equivalent to grade 1 procedures but also including some grade 2 procedures, delivery should be accomplished within 30 minutes where possible [1, 7, 8]. 

In clinical practice, adherence to this 30-minute timeframe often seems unachievable, or at least, is often not achieved [4, 5, 7]. In the National Sentinel Caesarean Section Audit (2001) only 63% of UK obstetric units were able to deliver even half of their most urgent cases within 30 minutes [9]. It has been suggested that longer decision-to-delivery times arise because a multitude of tasks has to be completed in a coordinated fashion by a relatively large multidisciplinary team before the caesarean can take place [10], thus, staff shortages, poor training, and lack of appropriate facilities all have the potential to slow the process.

A qualified midwife is a crucial member of the multidisciplinary team caring for any woman in need of a grade 1 or grade 2 caesarean, and this is part of the justification for UK maternity service policies stating that women in established labour should be provided with one-to-one midwifery care [11–14]. The Royal College of Midwives Annual Survey of UK Heads of Midwifery (2005) revealed, however, that almost 75% of maternity units experience staffing shortages and cannot provide this level of care [15, 16]. In the present study, we set out to measure the impact of workload and midwifery staffing levels on the length of time taken to transfer a woman to the operating theatre and ultimately to deliver her baby by grade 1 or grade 2 caesarean. Our secondary aims were to discover whether the time of day, the form of anaesthesia chosen for the procedure, the time spent in the operating theatre preparing the woman for her surgery, or the speed of surgery itself contributed significantly to delay in delivery of the baby.

## 2. Methods

This was a 12-month-long prospective audit of clinical practice carried out on the delivery suite of a tertiary referral hospital in the North of England in the UK in 2006. An audit proforma was completed by the Obstetrician performing the surgery in conjunction with the attending Midwife after every grade 1 and grade 2 caesarean performed during the study period. Forms were collated by the research team within 72 hours of the operation, and missing data were added to the forms at that time when necessary.

The primary indication for each procedure was recorded together with its designated grade, the time of decision, the time that the patient entered the operating theatre, the time that the operation was commenced, and the time that the baby was delivered. The form of anaesthesia used for each caesarean was also noted. Finally, the total number of labouring women present on the delivery suite at the time of the caesarean was recorded, in addition to the number of qualified midwives present on the shift.

Scatter plots were then constructed to demonstrate the relationships between the decision-to-delivery interval and the time of day, the number of labouring women and the Labouring Woman : Midwife (LW : MW) ratios on the delivery suite when the caesareans were being performed. Chi-squared analyses were used to compare categorical variables, and analysis of variance was used to compare groups of continuous variables, with two tailed *t*-tests used to make more detailed comparisons where appropriate. Values of *P* below 0.05 were taken to indicate statistical significance.

## 3. Results

### 3.1. Diurnal Variations in Practice

In the study year, 5581 women gave birth at the unit including 414 women who were delivered by elective caesarean section. Of the remaining 5167 women, 2620 gave birth between 08.31 hrs and 20.30 hrs, corresponding to junior doctors' daytime delivery suite shifts, with on-site cover provided by one Obstetric Registrar, one Obstetric Senior House Officer, one Anaesthetic Registrar, one Consultant Obstetrician, and one Consultant Anaesthetist. A further 2547 women gave birth between 20.31 hrs and 08.30 hrs, corresponding to junior doctors' night-time delivery suite shifts, with on-site cover provided by two Obstetric Registrars, one Obstetric Senior House Officer, and one Anaesthetic Registrar plus on-call and hence potentially off-site cover provided by one Consultant Obstetrician and one Consultant Anaesthetist. Two obstetric operating theatres were available throughout the day and night, staffed by an Anaesthetic Nurse or an Operating Department Assistant or Practitioner.

There were 755 emergency caesareans carried out in the study period. Of these, 122 were grade 1, 211 were grade 2, and 422 were grade 3 procedures. The mean decision-to-delivery interval for grade 1 caesareans was 23 minutes (SD 11) with 82.0% of babies being born within 30 minutes of the operation being requested and 99.2% being born within 75 minutes. For grade 2 caesareans, the mean decision-to-delivery interval was 32 minutes (SD 13) with 45.0% of babies being born within 30 minutes of the operation being requested and 98.1% being born within 75 minutes.

In all, the decision to perform a grade 1 caesarean was as likely to be made during the daytime as overnight (59/2620 versus 63/2547, *P* = 0.104) and the individually measured indications for grade 1 procedures did not differ between the two time frames (Table 1). By way of contrast, proportionately less grade 2 caesareans were performed during the day than during the night (97/2620 versus 114/2547, *P* = 0.015), and the greatest contributions to this difference came from a rise in the number of procedures being performed in response to a pathological CTG overnight without recourse to fetal blood sampling and a rise in procedures performed for nonprogressive labour in the second stage (Table 1).

**Figure 1:**
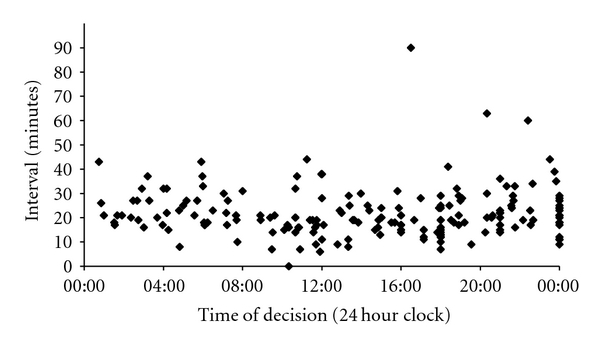
Decision-to-delivery interval versus time of day (grade 1).

**Figure 2:**
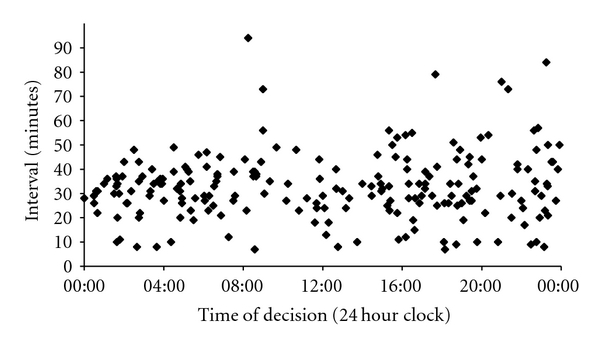
Decision-to-delivery interval versus time of day (grade 2).

Figures 1 and 2 demonstrate the variation in the decision-to-delivery interval during different times of the day. In our series, the time at which the decision was made to perform either a grade 1 or a grade 2 caesarean had no measurable impact on the amount of time it then took to deliver the baby.

### 3.2. Decision-to-Delivery Interval Related to the Number of Women in Active Labour and to the LW : MW Ratio

**Figure 3:**
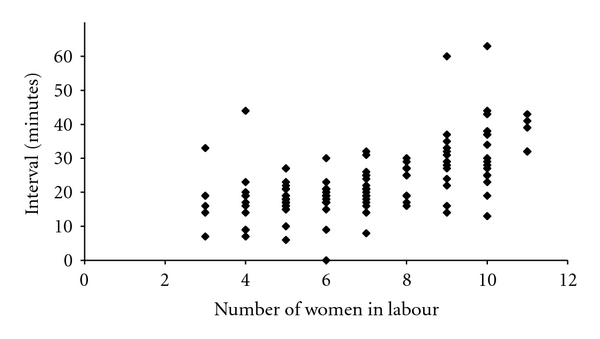
Decision-to-delivery interval versus number of labourers (grade 1).

**Figure 4:**
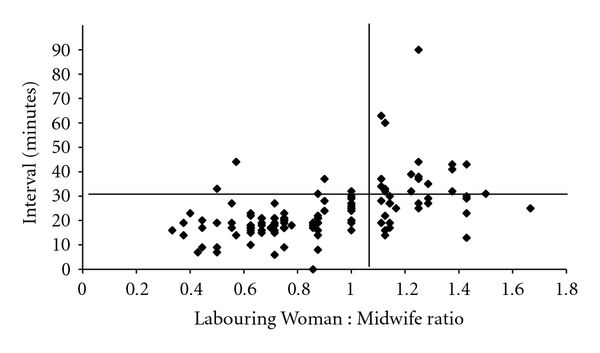
Decision-to-delivery interval versus Labouring Woman : Midwife ratio (grade 1).

For grade 1 caesareans the 30 minute cut-off was rarely breached until the number of laboring women on the delivery suit exceeded eight. Beyond this point, the 30-minute time limit was frequently exceeded (Figure 3). In keeping with this, when 1 : 1 care or better was being provided by midwives, 77/82 grade 1 caesareans were performed with a decision-to-delivery interval below 30-minutes but the time taken to deliver a baby increased significantly as the ratio deteriorated, so that only 22/40 babies were born within the 30 minute window when the LW : MW ratio was worse than 1 : 1 (*χ*_1_^2^, *P* < 0.001) (Figure 4).

**Figure 5:**
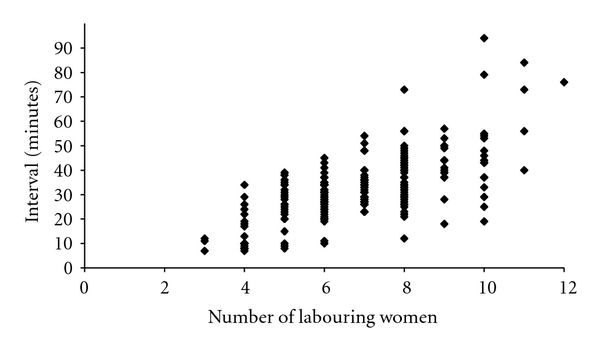
Decision-to-delivery interval versus number of labourers (grade 2).

**Figure 6:**
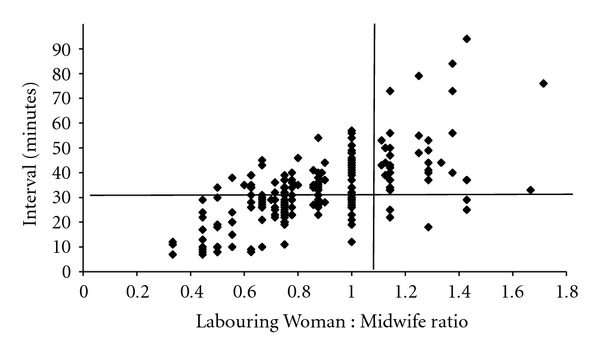
Decision-to-delivery interval versus Labouring Woman : Midwife ratio (grade 2).

For grade 2 caesareans, the decision-to-delivery interval was generally longer than for grade 1 procedures, and both the total number of women in active labour and the LW : MW ratio had a marked effect on the decision-to-delivery interval (Figures 5 and 6). When 1 : 1 care or better was being provided by midwives, 90/168 grade 2 caesareans were performed with a decision-to-delivery interval below 30 minutes but the time taken to deliver a baby increased significantly as the LW : MW ratio deteriorated, so that only 5/43 babies were born within the 30-minute window when the ratio fell below 1 : 1 (*χ*_1_^2^, *P* < 0.001).

### 3.3. Transfer Times to Theatre Related to the Number of Women in Active Labour and to the LW : MW Ratio

**Figure 7:**
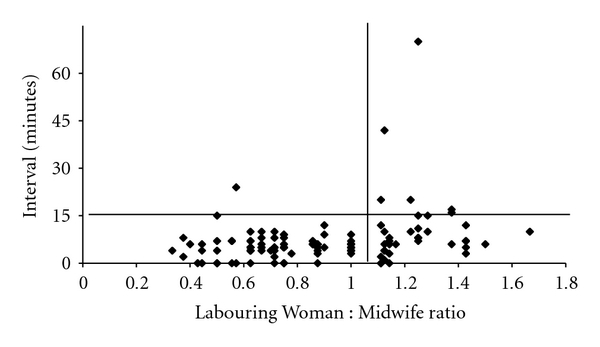
Transfer time to theatre versus Labouring Woman : Midwife ratio (grade 1).

For grade 1 caesareans the time taken to transfer the patient to theatre was more than 15 minutes in only a minority of cases but a deteriorating LW : MW ratio still had a measurable negative impact on transfer times (Figure 7). Thus, when 1 : 1 care or better was being provided by midwives, transfer to theatre was achieved within 15 minutes for 81/82 grade 1 caesareans but was only achieved for 34/40 grade 1 caesareans when the ratio fell below 1 : 1 (*χ*_1_^2^, *P* < 0.001).

**Figure 8:**
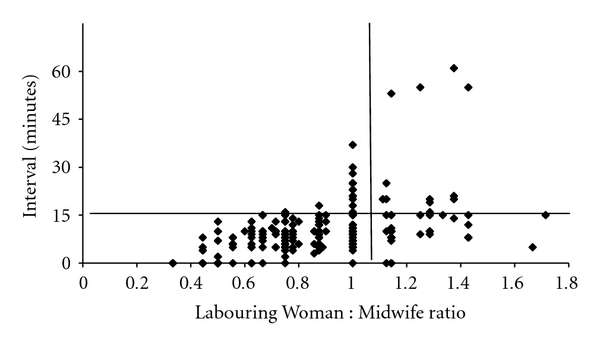
Transfer time to theatre versus Labouring Woman : Midwife ratio (grade 2).

Transfer of a patient to theatre for a grade 2 procedure was also influenced by the LW : MW ratio—when the ratio was 1 : 1 or better, transfer within 15 minutes was achieved in 155/168 cases whereas transfer within 15 minutes was only achieved in 29/43 cases when the LW : MW ratio was worse than 1 : 1 (*χ*_1_^2^, *P* < 0.001) (Figure 8).

### 3.4. Provision of Anaesthesia

Table 2 illustrates the forms of anaesthesia used in this case series. For grade 1 caesareans, general anaesthesia was more likely to be used in the daytime than overnight (31/59 versus 22/63, *P* = 0.005) whereas spinal blockade, became the most commonly used anaesthetic overnight (17/59 versus 29/63, *P* = 0.009). Comparing general anaesthesia, spinal blockade and epidural top-up, the choice of anaesthetic had a significant influence on the decision-to-delivery interval (ANOVA, *F* = 5.155, *P* = 0.007). We found that the mean decision-to-delivery interval for women having a grade 1 caesarean was 19.7 minutes (SD 8.5) under general anaesthesia, compared to 27.0 minutes (SD 8.2) under spinal blockade (*P* < 0.001). The decision-to-delivery interval was 26.0 minutes (SD 18.7) when the caesarean was performed under an epidural top-up, which was usually administered prior to the woman leaving her delivery room for the operating theatre.

For grade 2 caesareans, most women were delivered under regional blockade, and the choice of anaesthetic did not vary significantly with the time of day (Table 2). The mean decision-to-delivery interval for women having a grade 2 caesarean was not influenced significantly by the choice of anaesthetic (ANOVA, *F* = 0.505, *P* = 0.681), with women having an epidural top-up being delivered in an average of 29.2 minutes (SD 15.4), women having general anaesthesia being delivered in 30.1 minutes (SD 19.4), and women having spinal blockade being delivered in 34.7 minutes (SD 12.0).

### 3.5. Time Span from Arrival in Theatre to Commencement of the Operation and Then Delivery of the Baby

**Figure 9:**
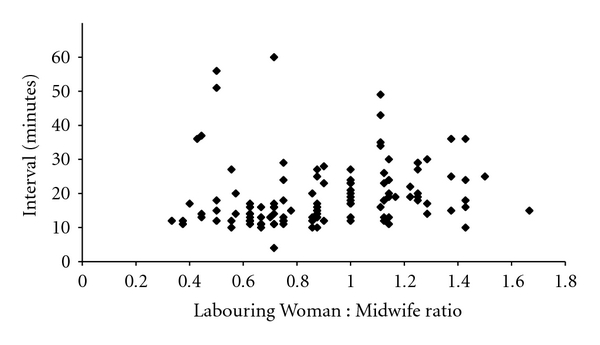
Preoperative time in theatre versus Labouring Woman : Midwife ratio (grade 1).

For women having a grade 1 caesarean, the mean time span from arrival in theatre to the start of the operation was 19.1 minutes (SD 9.6) but this time varied with the form of anaesthesia employed (ANOVA, *F* = 18.4, *P* < 0.001). If the caesarean was performed under general anaesthesia, the operation was commenced 14.4 minutes (SD 6.0) after the woman's arrival in theatre, which compared favourably to the mean time span of 24.6 minutes (SD 9.6) if a spinal blockade was used (*t*-test, *P* < 0.001) and to the mean time span of 20.0 minutes (SD 11.4) if an epidural top-up had been administered (*t*-test, *P* = 0.032).  Figure 9 demonstrates the relationship of the LW : MW ratio to the time taken from arrival of the patient in the operating theatre until commencement of the operation for grade 1 procedures. Although the midwife performs a number of essential steps in the preparation of a woman for caesarean in theatre, the LW : MW ratio had no discernible bearing on this time interval. 

**Figure 10:**
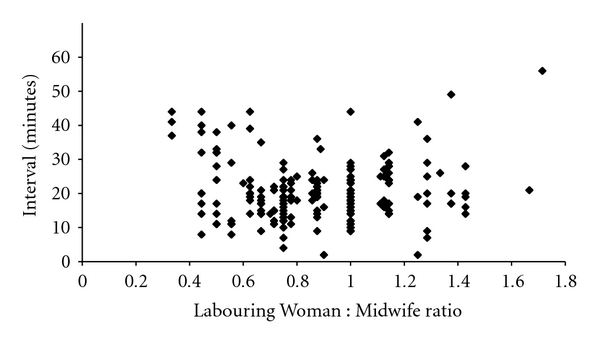
Preoperative time in theatre versus Labouring Woman : Midwife ratio (grade 2).

For women having a grade 2 caesarean, the mean time span from arrival in theatre to the start of the operation was 20.4 minutes (SD 8.6), which was not significantly different to the time span for grade 1 procedures (*t*-test, *P* = 0.201). If a general anaesthetic was administered, this phase lasted 18.2 minutes (SD 14.9) compared to 21.1 minutes (SD 6.5) if a spinal block was employed and 19.9 minutes (SD 10.3) if an epidural top-up had been given. Analysis of variance revealed no statistically significant difference in performance between these forms of anaesthesia (ANOVA, *F* = 1.10, *P* = 0.335).  Figure 10 demonstrates the relationship of the LW : MW ratio to the time take from arrival of the patient in the operating theatre until commencement of the operation for grade 2 procedures. Again, the LW : MW ratio had no discernible bearing on this time interval. 

The time taken from skin incision to the delivery of the baby was 3.3 minutes (SD 1.9) for grade 1 caesareans and 4.7 minutes (SD 3.1) for grade 2 caesareans, giving an average saving of 1 minute and 24 seconds for the more urgent procedures (*P* < 0.001).

## 4. Discussion

More than one in five UK babies is born by caesarean and of these approximately 63% are emergency procedures [9]. In our series, of the 755 emergency caesareans identified, 422 were grade 3 procedures with no evidence of maternal or fetal compromise. This paper focuses on grade 1 and 2 procedures, providing greater detail than what has previously been published about the timing of events between the decision to deliver a baby in such circumstances and the eventual birth of the baby. We have shown that for both grade 1 and grade 2 caesareans, delivery is most likely to be achieved within 30 minutes if the complement of qualified midwives on a delivery suite is sufficient to allow one-to-one care to be provided to women in active labour. More specifically, failure to provide this level of care hinders the woman's transfer to the operating theatre but, once the woman has arrived in theatre, the labouring woman to midwife ratio has no further bearing on the delivery time of the baby. The present study did not investigate why poor LW : MW ratios should delay transfer of a woman to theatre for her caesarean, but a multitude of tasks must be performed by the midwife before theatre transfer is possible, as most theatre check lists will attest. Any “away-time” that the midwife has from her Preoperative patient, for example, to provide clinical care to another woman or to arrange for a colleague to provide that care, is therefore likely to delay theatre transfer.

This phenomenon may be most relevant to the performance of grade 1 caesareans, when individual minutes rather than tens of minutes can be crucial to the well-being of a hypoxic fetus. Commendably, however, for grade 1 caesareans the effect of poor LW : WM ratios appears to be relatively small, possibly because midwifery and other staff prioritise care in order to achieve rapid transfer of the woman to theatre, preparation of the woman for surgery in the operating theatre, then actual delivery of the baby. As a result, in the present study, relatively few women having a grade 1 caesarean were delivered beyond the 30-minute benchmark.

For grade 2 caesareans, there was a clearer lengthening of the time that it took to transfer a woman to the operating theatre when LW : MW ratios deteriorated and, hence, a lengthening of the decision-to-delivery interval. This is of concern because although grade 2 caesareans are not performed when the life of the mother or fetus is in imminent danger, they are nonetheless performed because of evidence of maternal or fetal (usually fetal) compromise. It is logical to deduce that any systematic, unnecessary delay in achieving delivery of the baby for this population will cause clinical outcomes to deteriorate, an effect which we have now set out to measure with further detailed audit work.

The time spanning arrival of a woman in theatre and the start of her operation did not differ between grade 1 and grade 2 caesareans in this study, nor with the LW : MW ratios on the delivery suite. This may have been because women in theatre are generally attended by a full complement of midwifery, medical, nursing, and ancillary staff whatever the level of urgency involved. We did, however, find that the provision of a general anaesthetic rather than a regional blockade shortened the decision-to-delivery time for grade 1 procedures, largely by minimising delay in the operating theatre itself. The same effect was not seen when grade 2 caesareans were being performed, highlighting the fact that this was an observational study rather than a randomised, interventional study; for grade 2 caesareans, it seems likely that women being delivered under general anaesthesia were in some physical or psychological way dissimilar to women delivered with a regional blockade whereas, for grade 1 caesareans, such considerations may have been circumvented. 

Like most other obstetric units in the UK, the pattern of medical staffing during the daytime differed from that seen overnight on the study site. Overnight, the delivery suite gained the services of an extra Obstetric Registrar but the Consultant Obstetrician and Consultant Anaesthetist provided on-call rather than on-site cover. The data suggest that either because of this change or because of some other unmeasured factor, clinical decision-making changed overnight. Obstetrically, little difference was seen in the numbers of grade 1 caesareans performed or the reasons that they were performed. In contrast, an increase in the proportion of women having a grade 2 caesarean section was seen overnight, largely because of an increase in the number of procedures being performed for CTG abnormalities (without use of an FBS) or because of nonprogressive labour in the second stage. The possibility is that the presence of a Consultant Obstetrician on-site overnight might redress this imbalance, but a well-constructed intervention study would be required to prove the benefit. It seems likely, however, that the drive towards 24-hour on-site cover from Consultant Obstetricians in busy UK obstetric units will continue without such data ever being generated. In anaesthetic practice, too, there were measurable differences between daytime and night time care. In particular, a more liberal use of general anaesthesia for grade 1 caesareans was seen in the daytime, possibly because of the increased presence of Consultant Anaesthetists, although other unmeasured factors such as variations in the case mix could also have influenced the results of this study.

The data presented here were generated in a busy, tertiary level hospital. As such, the findings of the study may not be applicable to smaller obstetric units, “stand-alone” Midwifery Led Units or “alongside” Midwifery Led Units, which deal with different case mixes and which may face quite different organisational challenges.

Finally, this study focussed upon the timing of events during grade 1 and grade 2 caesareans and the influence that a number of factors could have on the various components of the decision-to-delivery interval. No data regarding maternal or fetal outcomes have been presented. Furthermore, the study was observational rather than interventional. Any conclusions drawn about the relative benefits of, for example, providing one-to-one midwifery care to labouring women and providing 24 hour on-site cover from Consultant Obstetricians and Consultant Anaesthetists would be speculative. The work does, nevertheless, provide greater detail than what has previously been published about the events leading to delivery by grade 1 or grade 2 caesarean, and it highlights areas of clinical practice worthy of more detailed appraisal.

## Figures and Tables

**Table 1:** Absolute numbers of women having grade 1 and grade 2 caesarean sections over a 12-month period. Indications are abbreviated as CTG (cardiotocographic abnormality with no fetal blood sample performed), FBS (low pH on fetal blood sampling), FTP1 (failure to progress in the first stage of labour), FTP2 (failure to progress in the second stage of labour), and Failed IVD (failed instrumental vaginal delivery, including forceps, ventouse or both). Denominator data for analyses included 2620 women giving birth in daytime hours (excluding elective caesarean sections) and 2547 women giving birth overnight.

	CTG	FBS	FTP1	FTP2	Failed IVD	Other
Grade 1 (day)	35	18	—	—	3	3
Grade 1 (night)	31	19	—	—	5	8
*χ*_1_^2^	*P* = 0.121	*P* = 0.136	Not applicable	Not applicable	*P* = 0.074	*P* = 0.019
Grade 2 (day)	37	18	6	11	10	15
Grade 2 (night)	62	11	3	20	9	9
*χ*_1_^2^	*P* < 0.001	*P* < 0.001	*P* = 0.024	*P* = 0.010	*P* = 0.142	*P* = 0.016

**Table 2:** Form of anaesthetic for grade 1 and grade 2 caesarean sections. Denominator data for analyses included 2620 women giving birth in daytime hours (excluding elective caesarean sections) and 2547 women giving birth overnight.

	Epidural top-up	Spinal blockade	General anaesthetic
Grade 1 (day)	11	17	31
Grade 1 (night)	12	29	22
*χ*_1_^2^	*P* = 0.610	*P* = 0.009	*P* = 0.005
Grade 2 (day) *n* =	27	63	7
Grade 2 (night) *n* =	33	70	11
*χ*_1_^2^	*P* = 0.108	*P* = 0.084	*P* = 0.079
